# Impact of program transfer from a non-governmental organization to a district health office: Evaluation of a program integrating water treatment and hygiene kits into reproductive health and HIV services, Machinga District, Malawi, 2010-2012

**DOI:** 10.1371/journal.pone.0219984

**Published:** 2019-07-31

**Authors:** Kirsten Fagerli, Janell Routh, W. Thane Hancock, Brooke Hoots, Andrews Gunda, Li Deng, Beth Tippett Barr, Mary Kamb, Robert Quick

**Affiliations:** 1 Division of Foodborne, Waterborne, and Environmental Diseases, National Center for Zoonotic and Emerging Infectious Diseases, Centers for Disease Control and Prevention, Atlanta, Georgia, United States of America; 2 National Center for HIV/AIDS, Viral Hepatitis, STD, and TB Prevention, Centers for Disease Control and Prevention, Atlanta, Georgia, United States of America; 3 Clinton Health Access Initiative, Lilongwe, Malawi; 4 Division of Global HIV and Tuberculosis, Center for Global Health, Centers for Disease Control and Prevention, Kisumu, Kenya; The Ohio State University, UNITED STATES

## Abstract

**Background:**

In September 2009, the Machinga Integrated Antenatal Water Hygiene Kit Program began addressing problems of unsafe water, high infant mortality, and low antenatal care (ANC) attendance in Machinga District, Malawi. In March 2011, the supporting international non-governmental organization transitioned management of the program to the Machinga District Health Office (DHO). We evaluated maternal and HIV service use before and after program transition to the DHO.

**Methods:**

We compared pre- and post-transition periods by examining data recorded in ANC and maternal registries in 15 healthcare facilities (HCFs) by proportion z-tests. We classified HCFs by size, using the median monthly patient volumes as the split for large or small facilities. We used logistic regression to evaluate changes in the use of ANC, maternal, and HIV services and their interactions with HCF size.

**Results:**

The percentage of women attending their first ANC visit during the first trimester was similar in the pre-and post-transition periods (9.3% vs 10.2%). Although the percentage of women with ≥4 ANC visits was similar from pre- to post-transition (26.0% vs 24.8%), the odds increased among women in small facilities (OR: 1.37, 95% CI: 1.24–1.51), and decreased among women in large facilities (OR: 0.80, 95% CI: 0.75–0.85). Although a similar percentages of pregnant women were diagnosed with HIV in all HCFs in the pre- and post-transitions periods (6.4% vs 4.8%), a substantially larger proportion of women were not tested for HIV in large HCFs (OR: 6.34, 95% CI: 5.88–6.84). A larger proportion of women gave birth at both small (OR: 1.30, 95% CI: 1.16–1.45) and large HCFs (OR: 1.55, 95% CI: 1.43–1.67) in the post-transition vs. the pre-transition period.

**Conclusions:**

The evaluation results suggest that many positive aspects of this donor-supported program continued following transition of program management from a non-governmental organization to a DHO.

## Background

Malawi has a high burden of maternal (675 per 100,000 live births) and infant (66 per 1,000 live births) mortality [1]. While a majority of pregnant women attend at least one antenatal care (ANC) visit, only 24% have their first ANC visit during the first trimester of pregnancy [2]. In 2015, only about half of all pregnant women completed the recommended four or more ANC (ANC4+) visits [2].

Inadequate use of ANC services throughout a pregnancy is associated with adverse health outcomes, such as maternal and perinatal mortality, low birth weight, and premature delivery [3–4]. In recent years, the government of Malawi and supporting international non-governmental agencies have prioritized efforts to increase the number of ANC clinic visits and healthcare facility (HCF) deliveries. As a result, the proportion of pregnant women in Malawi that received ANC from a skilled provider rose from 90% in 1992 to 95% in 2015 [2].

In 2010, only 66% of pregnant women attending antenatal clinics were tested for human immunodeficiency virus (HIV) [5], despite high HIV prevalence among women of reproductive age. Coverage of pregnant women in prevention of mother-to-child transmission (PMTCT) programs was 53% in 2010, well below the goal for 2013 of 80% set by the Malawi Ministry of Health (MOH) [6].

Improved maternal and child health, as well as comprehensive HIV services, were set as priority targets toward the achievement of Millennium Development Goals 4, 5, and 6 by the United Nations from 1990 to 2015 [7]. To address these goals, from September 2009 –March 2011, the Malawi MOH and Clinton Health Access Initiative (CHAI), an international non-governmental organization, developed a package of incentives to increase use of ANC, delivery, and HIV services. The incentives, called “water hygiene kits”–consisting of water storage container with a lid and tap, sodium hypochlorite water treatment solution, oral rehydration salts, and soap–were offered to rural-dwelling pregnant women at their first ANC visit, and refills of water treatment solution and soap were provided at subsequent ANC visits and delivery. At the time of this program, PMTCT Option B+ (provision of lifelong antiretroviral therapy to HIV-infected pregnant women), was implemented in Malawi. Because success of this program was heavily dependent on male partner participation [8–9], CHAI required partners’ presence for women to receive the initial water hygiene kit. During the 18-month program, CHAI distributed over 25,000 water hygiene kits as incentives to promote male involvement and family-centered ANC at 15 healthcare facilities in Machinga District.

At the time of the first ANC visit, HIV counseling and testing (HCT) services were offered to participating couples. Trained community volunteers living with HIV/AIDS provided counseling to individuals testing HIV-positive and assisted them to referral locations able to stage their infections and provide recommended PMTCT services including antiretroviral therapy (ART). All women were eligible to receive up to four free refills of sodium hypochlorite solution and soap during subsequent ANC visits, at delivery, and during postnatal checkups. An evaluation conducted 1 year into the project found increased ANC attendance, delivery at HCFs, household water treatment with sodium hypochlorite, safe storage of drinking water, and the ability of mothers to demonstrate proper handwashing technique [10]. The program exceeded expected levels of HCT participation among pregnant women and their partners.

Program transition, defined as the formal handing over of a program to local partners or the host government, is vital in sustaining key aspects of programs over the long-term [11]. While a number of studies based in high-income countries have documented the sustainability of program transition [12–13], few studies have examined the sustainability of program transition in low and middle-income nations [11]. In March 2011, CHAI transitioned the water hygiene kit program management to the Machinga District Health Office (DHO) over a 9-month period. During this period, all but one member of the CHAI team left the project, leaving one staff member to provide program guidance to DHO personnel. The transition consisted of handing over warehousing and management of water hygiene kit distribution to DHO personnel, providing project records to the DHO, ensuring that DHO staff continued scheduling quarterly HCF visits, and orienting the DHO statistician to management of ANC and delivery registry data, which included providing quarterly aggregated district reports and healthcare facility-specific reports to the DHO and district HCFs. In January 2012, we evaluated whether levels of ANC attendance, hospital delivery, and HIV service use were maintained following program transition.

## Methods

### Study site

The population of Machinga District in the Southern Region of Malawi has low educational levels and socioeconomic status, with only 5.6% of women completing primary school [1]. The district also has limited access to safe water and sanitation, with 77.8% of households using an improved water source, 16.8% of households using an effective water treatment method, and 10.1% of households having an improved, not shared, sanitation facility [1].

In addition, the district has a low percentage of women delivering in a healthcare facility (74.4%), and high rates of infant mortality (77 per 1,000 live births) compared to the country average (66 per 1,000 live births) [1]. In 2010, the prevalence of HIV in Malawi was 13.0 (11.9–13.5) for women 15–49 years old [14].

### Evaluation design

**Fig 1:**
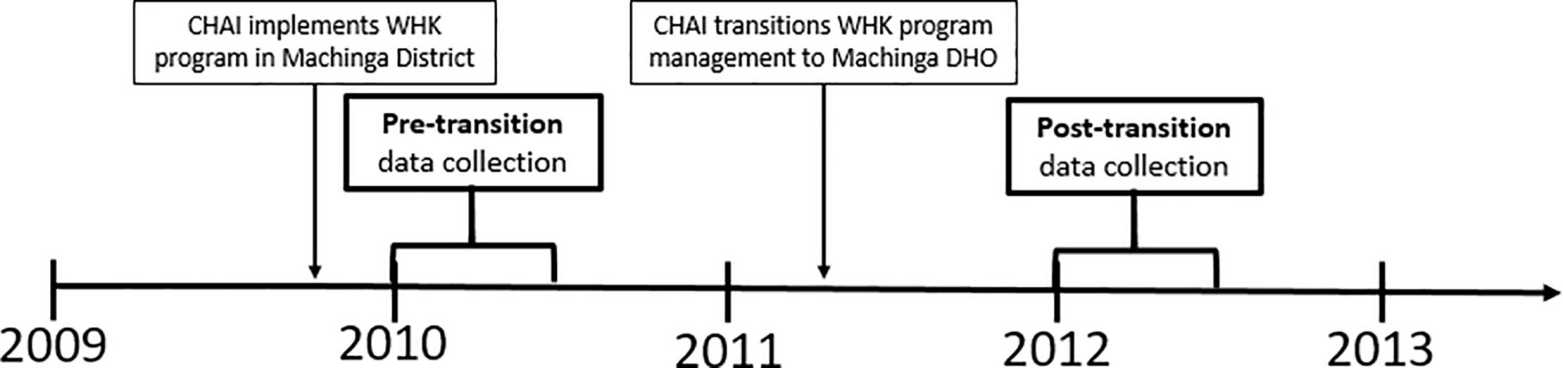
Timeline for the implementation and transition of the water hygiene kit program management from September 2009—June 2012.

In March 2011, CHAI initiated transition of the water hygiene kit program to the Machinga DHO. One CHAI project officer remained in Machinga to support the DHO during the transition (Fig 1). Water hygiene kits had been previously procured by CHAI and were stored in a hospital warehouse until they were ready to be distributed. To determine whether the program remained successful in motivating ANC and delivery service use, we compared post-transition antenatal and maternity registry data in 15 healthcare facilities from January–June 2012 to pre-transition registry data from January–June 2010, when a full CHAI implementation team was involved in all aspects of the project.

### Registry data collection

Data from antenatal and maternity registries were recorded at 15 participating HCFs during the pre-transition (January–June 2010) and post-transition periods (January–June 2012). For the cohorts of women whose first antenatal visit occurred during the January to June periods in 2010 (pre-transition cohort) and in 2012 (post-transition cohort), the antenatal registries showed aggregate data for outcome variables of interest. These variables included the number of first ANC visits during each of the two periods, the total number of ANC visits made during each of these periods, and HIV status determined during each of these periods. Maternity registry data were collected from mothers when they presented at a HCF at the time of delivery or within 72 hours of giving birth. Registry data included location of delivery and ART use during delivery.

### Statistical analysis

Data were analyzed using SAS version 9.4 (Cary, NC). We used 2-sample z-test to compare ANC and maternity registry outcomes between pre-transition and post-transition periods at all HCFs. The 15 facilities were grouped into “small” and “large” HCFs using the median 6-month aggregated pre-transition period patient volumes (post-transition patient volumes did not change sufficiently in any of the HCFs to change these groupings). We applied logistic regression models to test the interaction between changes in proportion of women using ANC and delivery services during the two periods and HCF size. Significant interactions were found in several outcome variables, implying the changes from pre- to post- transition periods depended on facility size. Then we evaluated the changes in ANC and delivery service use by HCF size (large vs. small) under logistic models. Women who had previously tested positive for HIV were excluded from the HIV analyses.

### Ethical considerations

The Human Subjects Advisor at the National Center for Emerging and Zoonotic Infectious Diseases at CDC determined that, because this activity consisted of an evaluation of a proven public health practice, did not use maternal interviews, and obtained no personal identifying data, it was exempt from human subjects research review (protocol #6082). The Malawi Ministry of Health authorized the evaluation and provided CHAI with a letter of approval for the evaluation of the comprehensive and integrated PMTCT pilot program in Machinga District.

## Results

ANC registries in the 15 HCFs included 14,935 women with at least 1 visit during the pre-transition period (January—June 2010), and 15,848 with at least 1 visit during the post-transition period (January—June 2012). Data from 1 month were missing for one of the 15 HCFs in the pre-transition period. The median number of women attending at least 1 ANC visit during the pre-transition period was 458 (interquartile range [IQR]: 417–719) at small HCFs (n = 7), and 1,267 (IQR: 1,096–1,654) at large HCFs (n = 8). During the post-transition period, the median number of women attending at least 1 ANC visit was 576 (IQR: 416–723) at small HCFs, and 1,378 (IQR: 1,111–1,782) at large HCFs.

During the pre-transition period, 8,796 women were seen at a maternity clinic within 72 hours of delivery, compared with 8,877 women during the post-transition period. One month of data was missing from one HCF in the pre-transition period and two HCFs in the post-transition period. The median number of new mothers seen in an HCF within 72 hours of delivery increased from pre- to post-transition periods in both small (269 [IQR: 211–409] vs 320 [IQR 275–429]), and large (737 [IQR: 564–1084] vs 780 [IQR: 583–1,029]) HCFs.

### First antenatal care (ANC1) visit during the first trimester

Overall, the percentage of women attending ANC1 during the first trimester was similar in the pre and post-transition periods (9.3% vs 10.2%) (Table 1). Comparing the pre-transition to post-transition period, there was an increase in the odds of pregnant women receiving their first ANC visit during the first trimester (odds ratio [OR]: 1.38, 95% confidence interval [CI]: 1.18–1.61) in small HCFs, and no difference in large HCFs (Table 2).

**Table 1:** Number and percentage of pregnant women with 4 or more antenatal (ANC4+) visits, first ANC (ANC1) visit in first trimester, and HIV service participation in 15 healthcare facilities in a prevention of mother to child HIV transmission program with water treatment and hygiene lit incentives before (January—June 2010) and after (January—June 2012) transition of management from a non-governmental organization to district supervision, Machinga District, Malawi.

		Number (%) with ANC1 visit during the first trimester of pregnancy				Number (%) of women with ANC4+ visits				Number (%) newly diagnosed with HIV(+) among women tested during current pregnancy				Number (%) of women not tested for HIV during current pregnancy			
	Facility	Pre-transition N (%)	Post-transition N (%)	% Change	p-value	Pre-transition N (%)	Post-transition N (%)	% Change	p-value	Pre-transition N (%)	Post-transition N (%)	% Change	p-value	Pre-transition N (%)	Post-transition N (%)	% Change	p-value
**Small Facilities**	**A**	6/252 (2.4)	75/416 (18.0)	15.6		65/252 (25.8)	154/416 (37.0)	11.2		19/208 (9.1)	30/368 (8.2)	-0.9		41/250 (16.4)	37/405 (9.1)	-7.3	
	**B**	36/450 (8.0)	63/394 (16.0)	8.0		79/450 (17.6)	156/394 (39.6)	22.0		26/351 (7.4)	33/344 (9.6)	2.2		88/440 (20.0)	44/388 (11.3)	-8.7	
	**C**	96/725 (13.2)	82/1157 (7.1)	**-6.1**		147/725 (20.3)	307/1157 (26.5)	6.3		39/640 (6.1)	41/973 (4.2)	-1.9		71/711 (10.0)	181/1154 (15.7)	**5.7**	
	**D**	49/688 (7.1)	49/657 (7.5)	0.4		247/688 (35.9)	173/657 (26.3)	**-9.6**		57/658 (8.7)	30/563 (5.3)	-3.4		25/683 (3.7)	78/641 (12.2)	**8.5**	
	**E**	45/719 (6.3)	79/723 (10.9)	4.6		101/719 (14.0)	201/723 (27.8)	13.8		39/655 (6.0)	30/675 (4.4)	-1.6		46/701 (6.6)	36/711 (5.1)	-1.5	
	**F**	10/458 (2.2)	52/538 (9.7)	7.3		222/458 (48.5)	258/538 (48.0)	-0.5		32/381 (8.4)	23/426 (5.4)	-3.0		68/449 (15.1)	90/516 (17.4)	2.3	
	**G**	37/417 (8.9)	50/576 (8.7)	-0.2		74/417 (17.7)	158/576 (27.4)	9.7		34/394 (8.6)	32/545 (5.9)	-2.7		20/412 (4.9)	18/563 (3.2)	-1.7	
**Large Facilities**	**H**	87/1378 (6.3)	127/1385 (9.2)	2.9		309/1378 (22.4)	265/1385 (19.1)	**-3.3**		50/1158 (4.3)	24/928 (2.6)	-1.7		214/1372 (15.6)	438/1366 (32.1)	**16.5**	
	**I**	84/1463 (5.7)	208/1855 (11.2)	5.5		360/1463 (24.6)	502/1855 (27.1)	2.5		78/1348 (5.8)	17/446 (3.8)	-2.0		106/1454 (7.3)	1404/1850 (75.9)	**68.6**	
	**J**	72/785 (9.2)	78/714 (10.9)	1.7		314/785 (40.0)	248/714 (34.7)	**-5.3**		59/780 (7.6)	46/629 (7.3)	-0.3		3/783 (0.4)	66/695 (9.5)	**9.1**	
	**K**	86/1089 (7.9)	102/889 (11.5)	3.6		301/1089 (27.6)	222/889 (25.0)	-2.7		49/1019 (4.8)	22/598 (3.7)	-1.1		52/1071 (4.9)	265/863 (30.7)	**25.8**	
	**L**	105/1103 (9.5)	99/1332 (7.4)	-2.1		252/1103 (22.8)	277/1332 (20.8)	-2.1		41/1049 (3.9)	24/911 (2.6)	-1.3		88/1097 (8.0)	411/1322 (31.1)	**23.1**	
	**M**	150/1845 (8.1)	148/1708 (8.7)	0.6		311/1845 (16.9)	327/1708 (19.1)	2.3		124/1647 (7.5)	54/1332 (4.1)	-3.4		186/1833 (10.2)	364/1696 (21.5)	**11.3**	
	**N**	447/2408 (18.6)	326/2133 (15.3)	**-3.3**		878/2408 (36.5)	473/2133 (22.2)	**-14.3**		149/2094 (7.1)	69/1219 (5.7)	-1.4		187/2381 (7.9)	877/2096 (41.8)	**33.9**	
	**O**	85/1155 (7.4)	82/1371 (6.0)	-0.6		227/1155 (19.7)	216/1371 (15.8)	**-3.9**		64/1008 (6.4)	42/907 (4.6)	-1.8		133/1141 (11.7)	441/1348 (32.7)	**21.0**	
**All facilities**		1395/14935 (9.3)	1620/15848 (10.2)	0.9	0.01	3887/14935 (26.0)	3937/15848 (24.8)	-1.2	0.02	860/13390 (6.4)	517/10864 (4.8)	-1.6	<0.001	1328/14778 (9.0)	4750/15614 (30.4)	21.4	<0.001

**Table 2:** Odds of changes in antenatal care indicators before (January—June 2010) and after (January–June 2012) transition of program management from a non-governmental organization to district supervision, by size of healthcare facility, Machinga District, Malawi.

	Small Healthcare Facility			Large Healthcare Facility		
	Pre-transition	Post-transition	Odds Ratio (95% CI)	Pre-transition	Post-transition	Odds Ratio (95% CI)
ANC registry	N = 3,709 (%)	N = 4,461 (%)		N = 11,226 (%)	N = 11,387 (%)	
ANC1 visit during the first trimester of pregnancy	279 (7.5)	450 (10.1)	1.38 (1.18–1.61)	1,116 (9.9)	1,170 (10.3)	1.04 (0.95–1.13)
≥4 ANC visits	935 (25.2)	1,407 (31.5)	1.37 (1.24–1.51)	2,952 (26.3)	2,530 (22.2)	0.80 (0.75–0.85)
Newly diagnosed HIV(+) among women tested during current pregnancy*	246/3,287 (7.5)	219/3,894 (5.6)	0.74 (0.61–0.89)	614/10,103 (6.1)	298/6,970 (4.3)	0.69 (0.60–0.80)
Not tested for HIV during current pregnancy*	359/3,646 (9.9)	484/4,378 (11.1)	1.14 (0.98–1.31)	969/11,132 (8.7)	4266/11,236 (38.0)	6.34 (5.88–6.84)

*Previous HIV(+) result not included in the analysis

### Four or more ANC visits (ANC4+)

Among 15 HCFs, the percentage of women with ANC4+ visits changed little from the pre-transition to post-transition period (26.0% vs 24.8%) (Table 1), although the amount of change varied by facility size. In small HCFs, comparing ANC coverage before and after transition, there was an increase in the odds of pregnant women with ANC4+ visits after transition (OR: 1.37, 95% CI: 1.24–1.51), but in large HCFs there was a decrease in women with ANC4+ after transition (OR: 0.80, 95% CI: 0.75–0.85) (Table 2).

### HIV testing

From the pre-intervention to post-intervention period, there were decreases in the odds of women who were tested for HIV. This was primarily observed in large HCFs (OR: 6.34, 95% CI: 5.88–6.84), with only a slight increase in small HCFs (OR: 1.14, 95% CI: 0.98–1.31) (Table 2).

Among those tested, the percentage of pregnant women newly diagnosed with HIV decreased modestly from the pre-transition to post-transition period (6.4% vs 4.8%) (Table 1). Comparing pre- to post-transition data, the odds of women newly diagnosed with HIV decreased slightly in both small (OR: 0.74, 95% CI: 0.61–0.89) and large (OR: 0.69, 95% CI: 0.60–0.80) HCFs (Table 2).

### Delivery in healthcare facilities

Among 15 HCFs, the percentage of women seen in a HCF within 72 hours of a delivery increased from 87.7% to 90.6% from pre- to post-transition period (Table 3). This increase was observed in both small (OR: 1.30, 95% CI: 1.16–1.45) and large facilities (OR: 1.55, 95% CI: 1.43–1.67) (Table 4).

**Table 3:** Number and percentage of women giving birth in healthcare facilities and receiving ART during labor in 15 healthcare facilities in a prevention of mother to child HIV transmission program with water treatment and hygiene kit incentives before and after (January—June 2012) transition of program management from a non-governmental organization to district supervision, Machinga District, Malawi.

		Mother delivered in a HCF				ART given during labor			
	Facility	Pre-transition N (%)	Post-transition N (%)	% Change	p-value	Pre-transition N (%)	Post-transition N (%)	% Change	p-value
**Small Facilities**	**A**	167/196 (85.2)	286/320 (89.4)	4.2		3/196 (1.5)	16/320 (5.0)	3.5	
	**B**	206/254 (81.1)	224/275 (81.5)	0.4		5/254 (2.0)	20/275 (7.3)	5.3	
	**C**	510/556 (91.7)	725/762 (95.1)	3.4		9/556 (1.6)	22/762 (2.9)	1.3	
	**D**	351/409 (85.8)	416/439 (94.8)	9.0		8/409 (2.0)	35/439 (8.0)	6.0	
	**E**	472/546 (86.5)	410/488 (84.0)	-2.5		21/546 (3.9)	35/488 (7.2)	3.3	
	**F**	176/211 (83.4)	176/229 (76.9)	-6.5		2/211 (2.8)	21/229 (9.2)	6.4	
	**G**	225/269 (83.6)	405/429 (94.4)	10.8		1/269 (0.4)	28/429 (6.5)	6.1	
**Large Facilities**	**H**	478/598 (79.9)	525/586 (89.6)	9.7		7/598 (1.2)	16/586 (2.7)	1.5	
	**I**	795/875 (90.9)	741/797 (93.0)	2.1		3/875 (0.3)	17/797 (2.1)	1.8	
	**J**	343/415 (82.7)	287/318 (90.3)	7.6		3/415 (0.7)	18/318 (5.7)	5.0	
	**K**	348/370 (94.1)	285/334 (85.3)	**-8.8**		8/370 (2.2)	14/334 (4.2)	2.0	
	**L**	522/571 (91.4)	575/580 (99.1)	7.7		2/571 (0.4)	9/580 (1.6)	1.2	
	**M**	1227/1275 (96.2)	1045/1127 (92.7)	**-3.5**		8/1275 (0.6)	70/1127 (6.2)	5.6	
	**N**	1110/1358 (81.7)	1047/1263 (82.9)	1.2		27/1358 (2.0)	74/1263 (5.9)	3.9	
	**O**	786/893 (88.0)	896/930 (96.3)	8.3		16/893 (1.8)	38/930 (4.1)	2.3	
**All facilities**		7716/8796 (87.7)	8043/8877 (90.6)	2.9	<0.001	127/8796 (1.4)	433/8877 (4.9)	3.5	<0.001

**Table 4:** Odds of changes in maternal health indicators before (January—June 2010) and after (January–June 2012) transition of program management from a non-governmental organization to district supervision, by size of healthcare facility, Machinga District, Malawi.

	Small Healthcare Facility			Large Healthcare Facility		
	Pre-transition	Post-transition	Odds Ratio (95% CI)	Pre-transition	Post-transition	Odds Ratio (95% CI)
Maternity registry	N = 2,124 (%)	N = 2,344 (%)		N = 6,672 (%)	N = 6,533 (%)	
Gave birth in a health facility	1,816 (85.5)	2,079 (88.7)	1.33 (1.12–1.59)	5,900 (88.4)	5,964 (91.3)	1.37 (1.22–1.54)
Received ART during labor	34 (1.6)	152 (6.5)	4.26 (2.93–6.21)	93 (1.4)	281 (4.3)	3.18 (2.51–4.03)

### ART during labor

The overall percentage of women given ART during labor increased from 1.4% to 4.9% from pre- to post-transition periods (Table 3). This observed increase was similar in both small (OR: 1.62, 95% CI: 1.26–2.08) and large (OR: 1.29, 95% CI: 1.10–1.52) HCFs (Table 4).

## Discussion

To our knowledge, this evaluation is the first to examine whether ANC, maternity, and HIV service delivery levels could be maintained following the transition from an incentives program administered by an NGO to local management by a Ministry of Health District Office. Although we found some positive and negative changes in service use for several outcomes in HCFs, the magnitude of change was less than 10% for most outcomes. The variations in magnitude and direction for several indicators merit examination.

### ANC1 visit in the first trimester

The proportion of women attending their first ANC visit in the first trimester increased in more than two thirds of HCFs from the pre-transition to post-transition periods. A possible explanation for this finding is that water hygiene kits motivated women to attend ANC visits earlier than normal. A previous evaluation of the CHAI program revealed that the improved water storage container, which was distributed in the water hygiene kit at the time of the first ANC visit, was particularly desirable [10]. It is possible that women were motivated to make their ANC1 visit earlier than the usual practice to ensure that they received their container. These findings suggest that the program was sufficiently maintained by the DHO during the post-transition period to sustain achievement of the objective of early initiation of antenatal care. To our knowledge, no other programs designed to increase ANC service were in progress in Machinga District during this project.

### ANC4+

Among the 15 facilities included in our analysis, we found a small decrease (26.0% to 24.8%) in the proportion of women with four or more ANC visits. However, disaggregating results by size of HCF identified an increase in the percentage of ANC4+ visits within small HCFs, whereas large HCFs had a small decrease. One possible explanation for this divergence in findings by size of clinic could be a result of changes in the ART program to incorporate Option B+ which required monthly clinic visits and decentralization of the program from large to smaller HCFs. Alternatively, it is possible that pregnant women attending HCFs with large patient volumes experienced longer waiting times or poor treatment by overburdened health workers—both of which have been documented in previous evaluations [15–17]. Other studies have suggested that economic incentives were insufficient to motivate women to increase their number of ANC visits [18]. An evaluation of this CHAI program was completed in 2011, and found that pregnant women and their partners valued the water hygiene kits, refills of water treatment solution, and soap; however, this evaluation did not examine how facility size or quality of care influenced these opinions [10]. The interaction of incentives and antenatal service use is complex and merits further research.

### HIV testing and treatment

In large HCFs, we found that the proportion of women that were not tested for HIV during their current pregnancy significantly increased from the pre- to post-transition periods. The increase could have resulted from large stock outs of HIV testing kits that occurred in early 2012 [9, 19]. Although the reasons for stock outs were not clear, a previous study examining the transition of a large scale HIV/AIDS prevention program from a donor-run program to local stakeholders similarly found substantial commodity stock outs post-transition as a result of changes to supply source and schedules [11]. The reality of commodity stock outs in a program’s post-transition period justifies an extended transition period to ensure the establishment of a functioning commodity forecasting and procurement system [11, 20]. Alternatively, it is possible that the proportion of women not tested for HIV increased as a result of a reduction in the number of staff testing women for HIV as the NGO transitioned control of the program over to the DHO. However, staffing records from the time of transition were not available to further investigate this theory.

### Delivery in HCFs

Our evaluation results suggest that maternity services in both small and large facilities were maintained from pre- to post-transition periods. However, this result could be the result of a MOH policy promoting delivery in HCFs [2]. The percentage of women in Malawi that delivered in an HCF increased from 57% in 2004, to 73% in 2010, to 91% in 2015–16 [1–2, 21]. These increases parallel similar findings in other countries that have instituted policies designed to increase healthcare facility delivery [18, 22], although increased patient volumes can also have the effect of decreasing service use [23]. The paradoxical decrease in ANC4+ visits and increase in deliveries in a HCF could also be explained, in part, by a common perception among pregnant women that labor and delivery pose greater health risks to mother and child than pregnancy, as noted in a previous study [24].

### ART during labor

In contrast to HIV testing data, the proportion of women who received ART during labor significantly increased in both small and large facilities from pre- to post-transition periods. These findings may be influenced by the implementation of Option B+, a program initiated by the MOH in 2011 in which all HIV-infected pregnant and breastfeeding women became eligible for lifelong ART [9]. Implementation of Option B+ preceded the transition period, which increased the need for a functioning procurement system [25].

### Limitations

This evaluation, which used programmatic data collected at the healthcare facility level, had several important limitations. First, because we examined surveillance data at the healthcare facility level, we were unable to directly link increases in, or maintenance of, ANC utilization, HCF deliveries, or HIV services with successful transition of the program from CHAI to the District Health Office. Other environmental changes occurring during the study interval (e.g., introduction of Option B+) may have accounted for some of the findings. Second, this evaluation had no true control group because financial and logistical constraints limited our analysis to registry data from Machinga District (and precluded data collection from other districts not participating in the program). Consequently, we were unable to definitively attribute evaluation results to the water hygiene kit intervention. Third, CHAI assigned one project officer to remain in the district to support the DHO during the post-transition period, who ensured water hygiene kits were procured and prepositioned and continuously available in the hospital warehouse. These activities were not sustainable long-term and may have exaggerated the actual impact of the program on service use. Fourth, because program implementation was limited to Machinga District, the results are not generalizable to other districts in Malawi or elsewhere. Fifth, this evaluation collected data for only 1 year after transition from CHAI to the Malawi District Health Office, limiting our ability to assess longer-term impact of the program’s management, as well as the potential sustainability of a program requiring external funding. Finally, we received anecdotal information that commodity stock outs of water hygiene kits, HIV testing kits, and other supplies were common throughout the evaluation period, which may have affected program impact on service use through loss of incentives.

### Conclusion

Low and middle-income countries are often faced with the challenge of maintaining health programs after external funding support is withdrawn, NGOs transition out of a district or country, or both. Results of this study suggest that program outcomes can be maintained following transition from intensive NGO support to full local management, particularly when supported by local health policy. Access to commodities is an important determinant of programmatic success; however, prepositioned commodities and the continued presence of one NGO staff member during this evaluation may have positively influenced these findings. In a world with limited development resources, it is important to continuously examine the determinants of program sustainability and consider how to maintain similar programs as they transition from NGO support to local management.

## Supporting information

S1 Data Minimal data set. (ZIP)
