# Supplementary material for: Impact of program transfer from a non-governmental organization to a district health office: Evaluation of a program integrating water treatment and hygiene kits into reproductive health and HIV services, Machinga District, Malawi, 2010-2012
Source: PLoS One. 2019 Jul 31;14(7):e0219984. doi: 10.1371/journal.pone.0219984 (PMC6668799; doi:10.1371/journal.pone.0219984)
Supplement: S1 Data — (ZIP) [file pone.0219984.s001.zip › DataDictionary_MalawiStudy_PlosOne_July2019.docx]

**BL_FU= 0 (Baseline), 1 (Follow-up)**

**Fac_size** – Facility size indicator using median patient volume at baseline as the split (1: large; 0: small)

**2010 (BL)/2012 (FU)**

(MATERNITY DATA)

**Facility**- health facility name

**HIV_prev_neg-** Had previous HIV negative result

**HIV_prev_pos**- Had previous HIV positive result

**HIV_new_neg-** Newly tested HIV neg

**HIV_new_pos**-Newly tested HIV pos

**no_test** - Have no HIV test past/current

**all_HIV-** Total number of women with HIV stats (Q1-5)

**all_HIV_pos** – Total women in the HF positive for HIV

**ART_labor**- Had ART during labour

**deliv_fac**- delivered this health facility (where clinic tabulating)

**deliv_transit**- delivered in transit to HF

**deliv_other_fac** - devliered at a HF other than the one where woman counted

**deliv_home**- delivered at home

*(ANC DATA)*

**Facility**- health facility name

**ANC1**- woman had only 1 ANC visit

**ANC2**- woman had 2 ANC visits

**ANC3** - woman had 3 ANC visits

**ANC4** - woman had 4 ANC visits

**ANC5** - woman had 5 or more ANC visits

**all_ANC** - total number of women in cohort *(should match “REF”, but in most cases doesn’t)*

**first_visit** - first visit occurred within first 12 weeks of pregnancy

**SP_no** -Received a total of 0 SP doses

**SP_yes** - Received a doses

**FeFo_tabs120 -**Received 120+ FeFo tablets

**syp_neg** -Syphilis test negative

**syp_pos-** Syphilis test positive

**syp_unk-** Syphilis test unknown

**NVP-** NVP syrup was dispensed for the baby
